# Poly[tris­(2-amino­butan-1-ol)copper(II) [hexa­kis-μ_2_-cyanido-κ^12^*C*:*N*-tetra­copper(I)] bis­(2-amino­butan-1-olato)aqua­copper(II) monohydrate]

**DOI:** 10.1107/S2414314624008459

**Published:** 2024-08-30

**Authors:** Peter W. R. Corfield, Paul Salvi

**Affiliations:** a Department of Chemistry and Biochemistry Fordham University, 441 East Fordham Road Bronx NY 10458 USA; Benemérita Universidad Autónoma de Puebla, México

**Keywords:** crystal structure, mixed valence Cu, CuCN network, 2-amino-1-butanol

## Abstract

The title structure, {[Cu(C_4_H_11_NO)_3_][Cu_4_(CN)_6_]·[Cu(C_4_H_10_NO)_2_(H_2_O)]·H_2_O}_*n*_, is made up of diperiodic honeycomb Cu^I^CN networks built from [Cu_4_(CN)_6_]^2−^ units, together with two independent Cu^II^ complexes: six-coord­inate [Cu(CH_3_CH_2_CH(NH_2_)CH_2_OH)_3_]^2+^ cations, and five-coordinate [Cu(CH_3_CH_2_CH(NH_2_)CH_2_O)_2_·H_2_O] neutral species. The two Cu^II^ complexes are not covalently bonded to the Cu^I^CN networks. Strong O—H⋯O hydrogen bonds link the Cu^II^ complexes into pairs and the pairs are hydrogen bonded into chains along the crystallographic *b* axis *via* the hydrate water mol­ecule. In addition, O—H⋯(CN) and N—H⋯(CN) hydrogen bonds link the cations to the CuCN network. In the honeycomb polymeric moiety, all bridging cyanido ligands are disordered over two orientations, head-to-tail and tail-to-head, with occupancies for C and N atoms varying for each CN group.

## Abstract 2

The title structure is made up of diperiodic anionic Cu^I^CN networks and two independent Cu^II^ complexes that are not covalently bonded to the networks.

## Graphical abstract

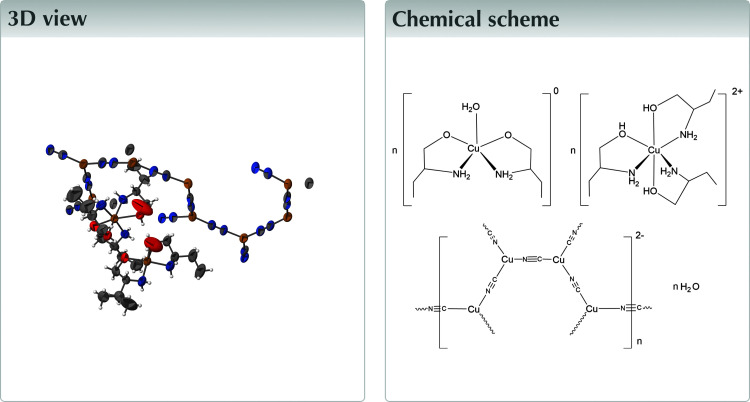

## Structure description

Copper cyanide networks are of continuing inter­est because of the wide variety of different networks found (Pike, 2012 ▸; Iwai *et al.*, 2023 ▸) and the inter­esting and potentially useful magnetic or photoluminescent properties shown by some of them (*e.g*. Lim *et al.*, 2008 ▸). Anionic Cu^I^CN networks, which are hosts to cationic conjugate acids of various amine bases, have been studied in order to understand how the various network structures relate to the nature of the hosted cations – the so-called template effect – and to investigate certain physical properties of the network structures (*e.g*. Pretsch & Hartl, 2004 ▸; Corfield *et al.*, 2022 ▸). There are fewer structurally characterized mixed-valence organic CuCN networks in the literature. Our previous work in this area has involved attempts to synthesize neutral CuCN networks that fully incorporate both Cu^I^ and Cu^II^ atoms (Corfield *et al.*, 2024 ▸; Corfield & Sabatino, 2017 ▸).

**Figure 1:**
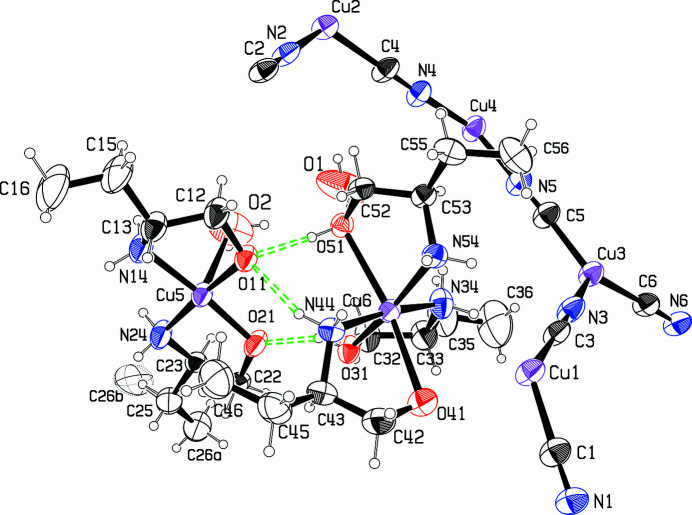
The asymmetric unit of the title compound, with 30% displacement ellipsoids. Colours are: C, H black; O red; N blue; Cu magenta. H atoms associated with the disordered methyl group C26*b* are omitted for clarity. Hydrogen bonds linking the Cu^II^ complexes together are shown as green dashed lines. H41 is not visible, as it is located behind O41 in this figure.

The title compound was obtained serendipitously during attempts to continue syntheses of these mixed-valence CuCN networks with the use of the base 2-amino-1-butanol. Instead of the expected structure type, with CN bridging Cu^I^ and Cu^II^ atoms, we obtained the title compound, where a Cu^I^CN network is host to guest Cu^II^ complexes. The asymmetric unit shown in Fig. 1 ▸ is comprised of Cu^I^ atoms (Cu1 to Cu4), Cu^II^ atoms (Cu5 and Cu6), six bridging cyanido ligands, five 2-amino-1-butanol bases, and two water mol­ecules, O2 coord­inated to Cu5 and O1 situated separately.

Of the two Cu^II^ atoms, Cu5 is coordinated by the two bases O11⋯C16 and O21⋯C26, as well as by a water mol­ecule, in a square-pyramidal arrangement with the H_2_O in the apical position, at a distance of 2.582 (12) Å. The bases have both lost their hy­droxy protons, making this complex neutral in charge. The bases are in the *cis* position relative to each other, and the chelated conformations are both λ. Atom Cu6 is coordinated by three chelating bases that have all kept their OH protons, so that this complex has a +2 charge. The chelates are all in the λ conformation. The coordination around Cu6 is elongated octa­hedral. Base O31⋯C36 coordinates in the equatorial plane, while bases O41⋯C46 and O51⋯C56 have their NH_2_ groups in the equatorial plane, and their OH groups in the axial positions, with long Cu—O axial bonds, at 2.508 (6) and 2.453 (5) Å. Bond Cu—O31 in the equatorial plane is much shorter at 1.956 (4) Å, although this distance is longer than the Cu—O distances of 1.901 (4) and 1.904 (4) Å in the Cu5 complex, where the H atoms have been lost. The equatorial Cu—N bond lengths in the octa­hedral complex of Cu6 average 2.022 (4) Å, slightly longer than those in the square-pyramidal Cu5 complex, which average 1.989 (5) Å.

**Figure 2:**
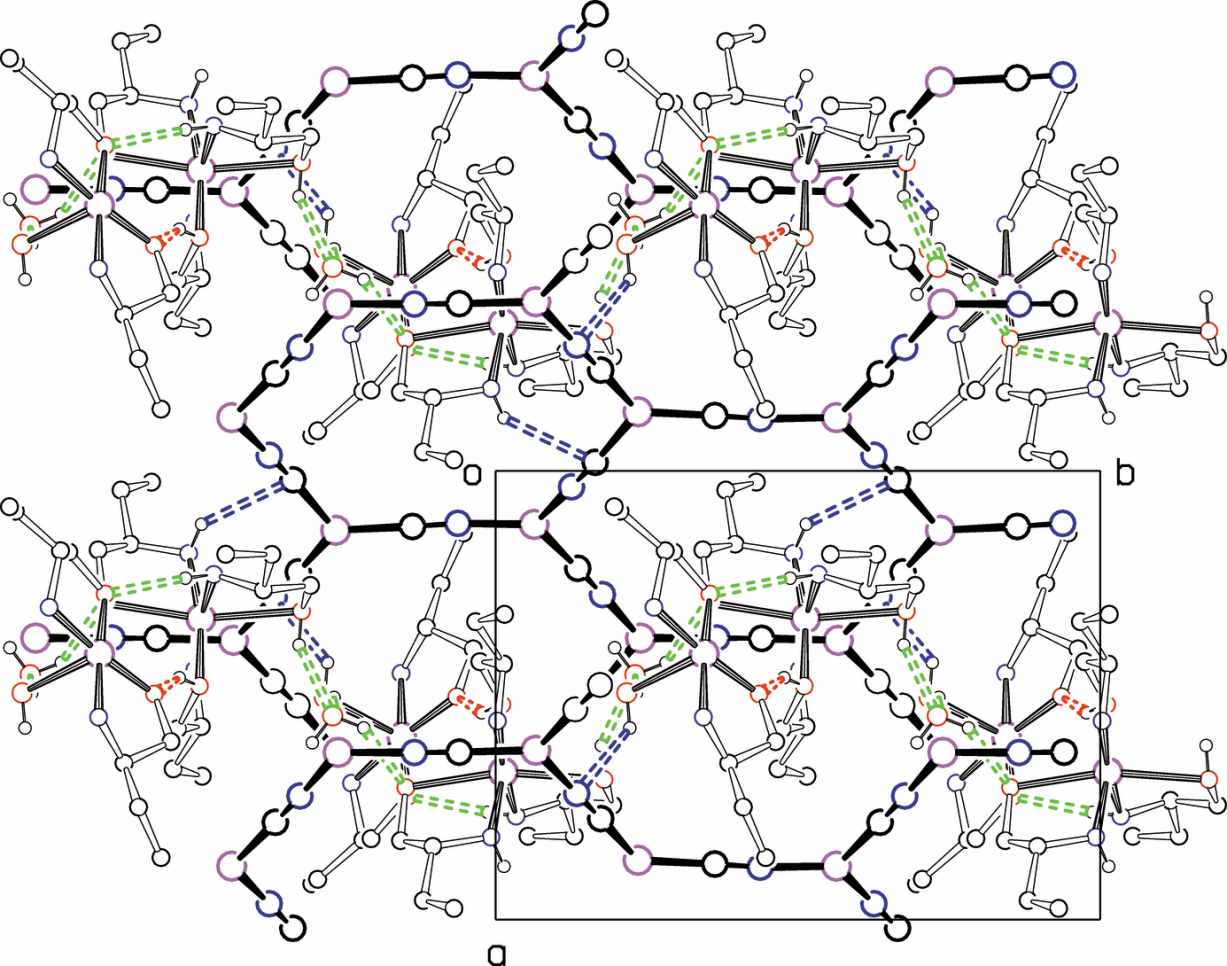
Packing diagram, showing the structure viewed down the *c* axis. Colours as in Fig. 1 ▸; All H atoms and C26*b* (disordered meth­yl) are omitted. Hydrogen bonds between the Cu^II^ complexes are shown as dashed green bonds, while those between the complexes and CN groups of the polymeric network are shown in blue.

Hydrogen bonds are listed in Table 1 ▸. The two Cu^II^ complexes are linked together by the short hydrogen bonds O31—H31⋯O21 and O51—H51⋯O11, as shown in Fig. 1 ▸. A somewhat longer hydrogen bond, N44—H44*B*⋯O11, also links the two complexes. Hydrogen bonding to the lattice water mol­ecule O1 links the pairs of Cu^II^ complexes into a chain along the *b* axis. These hydrogen bonds are also shown in the packing diagrams, Figs. 2 ▸ and 3 ▸. Two hydrogen bonds link the pairs of complexes to the CuCN network, and these are shown in blue in Figs. 2 ▸ and 3 ▸. There may be other weaker inter­actions with the network, but their distances are outside the 3.2 Å limit that we set.

The [Cu_4_(CN)_6_]^2−^ units making up the diperiodic network form planar honeycomb networks made up of 18-membered CuCN rings, parallel to plane (101) in the crystal. Each of the four independent Cu atoms involved is close to coplanar with its three coordinated CN groups, with maximum deviation of Cu atom from its neighbours of 0.068 (4) Å for Cu4. Each of these Cu atoms is distorted from trigonal planar coordination in the same way: one of the three bond angles at Cu is larger, average 128.1 (7)°, than the other two, which average 115.9 (10)° (standard deviations given are of the mean). The average Cu—(C/N) distance for the two bonds surrounding the larger angle is slightly shorter than the third Cu—(C/N) bond length. The angle distortions lead to the 18-membered CuCN rings being somewhat lengthened in the direction of the screw axes.

The first organic CuCN complex described in the literature (Williams *et al.*, 1972 ▸) had a similar mixed-valence structure to the one described here. In that case, a three-dimensional Cu^I^CN network hosts guest [Cu(en)_2_H_2_O]^2+^ cations, where en = ethyl­enedi­amine. In a search of the Cambridge Structural Database (CSD, Version 5.35; Groom *et al.*, 2016 ▸), we found relatively few other organic CuCN network structures of this type. Entries COXRIR (Benmansour *et al.*, 2009 ▸) and COXRIR01 (Etaiw *et al.*, 2015 ▸) describe a diperiodic Cu^I^CN network hosting Cu(en)_2_ cations, and entry UGUTOF (Colacio *et al.*, 2002 ▸) describes a three-dimensional Cu^I^CN network with guest Cu^II^ cations coordinated by 2-methyl­ethylenedi­amine. There are also three inorganic CuCN networks with guest [Cu(NH_3_)_4_]^2+^ cations.

## Synthesis and crystallization

A mixture of 5.02 mmol CuCN and 8.12 mmol NaCN was added to 20 ml of H_2_O and stirred until all the mixture had dissolved. In a separate container, 10.06 mmol of 2-amino-1-butanol were dissolved in 10 ml H_2_O and added to the solution while stirring under heat. The solution immediately developed a faint purple tint. The pH was 11.9. The beaker was covered and allowed to sit for approximately 72 h, after which point a heterogeneous mixture of navy blue crystals and pale blue material was recovered. The structure presented here is based upon diffraction data from one of the dark blue crystals. IR spectra (cm ^−1^): 2117 (*s*), (CN stretch); 3440 (*versus*, broad) (O—H) stretch; 3328 (*sh*), 3272 (*sh*) (N—H stretch). We have not identified any sharp OH peak that might be expected for the strong O—H ⋯ O hydrogen bonds in the structure.

## Refinement

Crystal data, data collection and structure refinement details are summarized in Table 2 ▸. Only reflections with a resolution up to 0.80 Å were used in the refinement, as the data in the shell beyond this had just 14% of reflections with *I* > 2σ(*I*). C- and N-bound H atoms were fixed in their expected positions, while O-bound H atoms were refined, with restraints. N-bound H atoms were fixed because refinements of these atoms did not provide any more satisfactory geometry. Their initial placement was facilitated by use of difference maps based upon low order data, and by the *SHELXL* HFIX 83 instruction (Sheldrick, 2015 ▸). The terminal CH_3_ group of the ethyl group in base O21⋯C26 is modelled as disordered between two possible orientations, with occupancies 0.615 (19) and 0.385 (19). In the polymeric part, all bridging cyano ligands were modelled over two orientations, head-to-tail and tail-to head, since this ligand, while coordinating Cu^I^, has no strong preference for any orientation. Both atomic sites in each C≡N group is then a mixture of C and N atoms. Atoms sharing the same site were constrained to have the same coordinates and displacement parameters, and their occupancies were fixed or refined using free variables: 0.5/0.5 for C1≡N1, 0.69 (8)/0.31 (8) for C2≡N2, 0.70 (8)/0.30 (8) for C3≡N3, 0.65 (8)/0.35 (8) for C4≡N4, 0.5/0.5 for C5≡N5 and 0.79 (8)/0.21 (8) for C6≡N6.

## Supplementary Material

Crystal structure: contains datablock(s) I. DOI: 10.1107/S2414314624008459/bh4088sup1.cif

Structure factors: contains datablock(s) I. DOI: 10.1107/S2414314624008459/bh4088Isup2.hkl

CCDC reference: 2379862

Additional supporting information:  crystallographic information; 3D view; checkCIF report

## Figures and Tables

**Figure 3:**
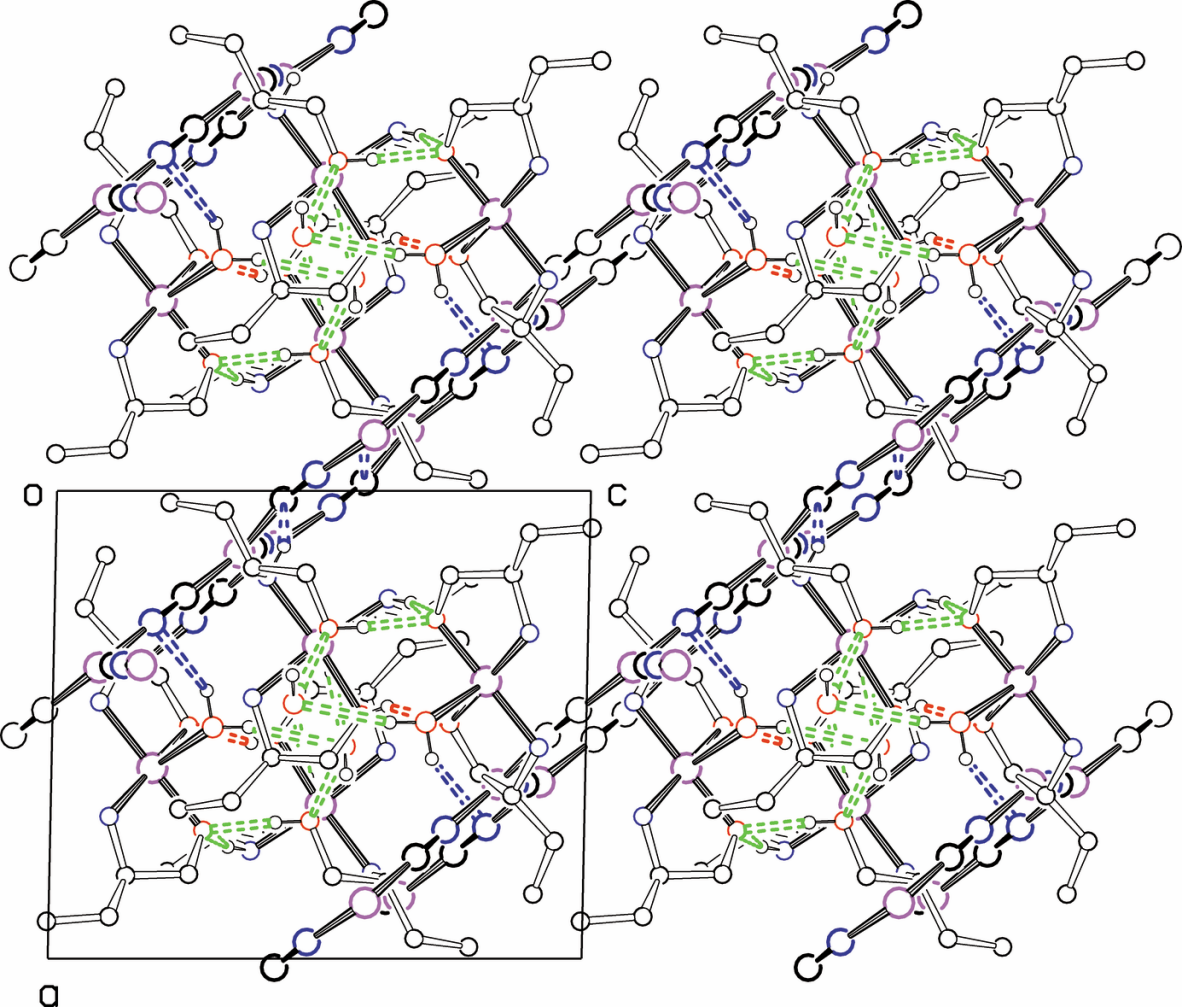
Packing diagram, showing the structure viewed down the *b* axis. Colours and hydrogen bonds as in Fig. 1 ▸. The CuCN honeycomb networks are seen edge-on in this projection.

**Table 1:** Hydrogen-bond geometry (Å, °)

*D*—H⋯*A*	*D*—H	H⋯*A*	*D*⋯*A*	*D*—H⋯*A*
O31—H31⋯O21	0.80 (3)	1.66 (2)	2.393 (6)	152 (4)
O51—H51⋯O11	0.83 (2)	1.74 (3)	2.562 (6)	174 (5)
N44—H44*B*⋯O11	0.89	2.10	2.950 (7)	159
O41—H41⋯O1^i^	0.86 (3)	1.93 (5)	2.726 (10)	154 (9)
N54—H54*A*⋯C2^ii^	0.89	2.51	3.180 (7)	133
O1—H1*A*⋯O51	0.84 (3)	1.93 (10)	2.669 (9)	146 (16)
O2—H2*B*⋯N3^iii^	0.89 (3)	2.47 (12)	3.125 (13)	130 (13)

Symmetry codes: (i) 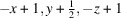; (ii) 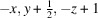; (iii) 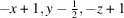.

**Table 2:** Experimental details

Crystal data	
Chemical formula	[Cu(C_4_H_11_NO)_3_][Cu_4_(CN)_6_]·[Cu(C_4_H_10_NO)_2_(H_2_O)]·H_2_O
*M*_r_	1017.06
Crystal system, space group	Monoclinic, *P*2_1_
Temperature (K)	297
*a*, *b*, *c* (Å)	11.1008 (2), 14.9561 (3), 12.7221 (2)
β (°)	91.486 (1)
*V* (Å^3^)	2111.47 (7)
*Z*	2
Radiation type	Mo *K*α
μ (mm^−1^)	3.02
Crystal size (mm)	0.33 × 0.30 × 0.04

Data collection	
Diffractometer	Enraf–Nonius KappaCCD
Absorption correction	Multi-scan (*SADABS*; Krause *et al.*, 2015 ▸)
*T*_min_, *T*_max_	0.47, 0.62
No. of measured, independent and observed [*I* > 2σ(*I*)] reflections	41170, 8587, 6363
*R*_int_	0.038
(sin θ/λ)_max_ (Å^−1^)	0.625

Refinement	
*R*[*F*^2^ > 2σ(*F*^2^)], *wR*(*F*^2^), *S*	0.030, 0.094, 1.13
No. of reflections	8587
No. of parameters	488
No. of restraints	56
H-atom treatment	H atoms treated by a mixture of independent and constrained refinement
Δρ_max_, Δρ_min_ (e Å^−3^)	0.53, −0.36
Absolute structure	Flack *x* determined using 2729 quotients [(*I*^+^)−(*I*^−^)]/[(*I*^+^)+(*I*^−^)] (Parsons *et al.*, 2013 ▸)
Absolute structure parameter	−0.002 (6)

Computer programs: *KappaCCD Server Software* (Nonius, 1997 ▸), *DENZO* and *SCALEPACK* (Otwinowski & Minor, 1997 ▸), *SHELXS97* (Sheldrick, 2008 ▸), *SHELXL2019/2* (Sheldrick, 2015 ▸), *ORTEPIII* (Burnett & Johnson, 1996 ▸) and *publCIF* (Westrip, 2010 ▸).

## full crystallographic data

### Poly[tris(2-aminobutan-1-ol)copper(II) [hexakis-µ2-cyanido-κ12C:N-tetracopper(I)] bis(2-aminobutan-1-olato)aquacopper(II) monohydrate] Crystal data

**Table:**

[Cu(C_4_H_11_NO)_3_][Cu_4_(CN)_6_]·[Cu(C_4_H_10_NO)_2_(H_2_O)]·H_2_O	*F*(000) = 1040
*M**_r_* = 1017.06	*D*_x_ = 1.600 Mg m^−^^3^
Monoclinic, *P*2_1_	Mo *K*α radiation, λ = 0.7107 Å
*a* = 11.1008 (2) Å	Cell parameters from 5040 reflections
*b* = 14.9561 (3) Å	θ = 1.0–27.5°
*c* = 12.7221 (2) Å	µ = 3.02 mm^−^^1^
β = 91.486 (1)°	*T* = 297 K
*V* = 2111.47 (7) Å^3^	Plate, blue
*Z* = 2	0.33 × 0.30 × 0.04 mm

### Poly[tris(2-aminobutan-1-ol)copper(II) [hexakis-µ2-cyanido-κ12C:N-tetracopper(I)] bis(2-aminobutan-1-olato)aquacopper(II) monohydrate] Data collection

**Table:**

Enraf–Nonius KappaCCD diffractometer	8587 independent reflections
Radiation source: fine-focus sealed tube	6363 reflections with *I* > 2σ(*I*)
Graphite monochromator	*R*_int_ = 0.038
Detector resolution: 9 pixels mm^-1^	θ_max_ = 26.4°, θ_min_ = 2.7°
combination of ω and φ scans	*h* = −13→13
Absorption correction: multi-scan (SADABS; Krause *et al.*, 2015)	*k* = −18→18
*T*_min_ = 0.47, *T*_max_ = 0.62	*l* = −15→15
41170 measured reflections	

### Poly[tris(2-aminobutan-1-ol)copper(II) [hexakis-µ2-cyanido-κ12C:N-tetracopper(I)] bis(2-aminobutan-1-olato)aquacopper(II) monohydrate] Refinement

**Table:**

Refinement on *F*^2^	Secondary atom site location: difference Fourier map
Least-squares matrix: full	Hydrogen site location: mixed
*R*[*F*^2^ > 2σ(*F*^2^)] = 0.030	H atoms treated by a mixture of independent and constrained refinement
*wR*(*F*^2^) = 0.094	*w* = 1/[σ^2^(*F*_o_^2^) + (0.0396*P*)^2^ + 0.587*P*] where *P* = (*F*_o_^2^ + 2*F*_c_^2^)/3
*S* = 1.13	(Δ/σ)_max_ < 0.001
8587 reflections	Δρ_max_ = 0.53 e Å^−^^3^
488 parameters	Δρ_min_ = −0.36 e Å^−^^3^
56 restraints	Absolute structure: Flack *x* determined using 2729 quotients [(*I*^+^)-(*I*^-^)]/[(*I*^+^)+(*I*^-^)] (Parsons *et al.*, 2013)
Primary atom site location: structure-invariant direct methods	Absolute structure parameter: −0.002 (6)

### Poly[tris(2-aminobutan-1-ol)copper(II) [hexakis-µ2-cyanido-κ12C:N-tetracopper(I)] bis(2-aminobutan-1-olato)aquacopper(II) monohydrate] Fractional atomic coordinates and isotropic or equivalent isotropic displacement parameters (Å^2^)

**Table:**

	*x*	*y*	*z*	*U*_iso_*/*U*_eq_	Occ. (<1)
Cu1	0.13212 (7)	0.73559 (5)	0.34410 (6)	0.0649 (2)	
Cu2	0.11909 (7)	0.06380 (5)	0.40774 (6)	0.0615 (2)	
Cu3	0.37743 (8)	0.56666 (6)	0.08561 (6)	0.0665 (2)	
Cu4	0.37261 (8)	0.23970 (6)	0.16423 (6)	0.0702 (3)	
Cu5	0.40743 (6)	0.34502 (6)	0.81097 (5)	0.0576 (2)	
Cu6	0.32863 (6)	0.51268 (6)	0.49443 (5)	0.05431 (19)	
C1	0.1237 (5)	0.8626 (5)	0.3695 (5)	0.0665 (16)	0.5
N1	0.1171 (6)	0.9375 (5)	0.3827 (5)	0.0643 (16)	0.5
C1N	0.1237 (5)	0.8626 (5)	0.3695 (5)	0.0665 (16)	0.5
N1C	0.1171 (6)	0.9375 (5)	0.3827 (5)	0.0643 (16)	0.5
C2	−0.0198 (5)	0.1648 (4)	0.5751 (5)	0.062 (2)	0.69 (8)
N2	0.0352 (6)	0.1255 (4)	0.5153 (6)	0.064 (2)	0.69 (8)
C2N	−0.0198 (5)	0.1648 (4)	0.5751 (5)	0.062 (2)	0.31 (8)
N2C	0.0352 (6)	0.1255 (4)	0.5153 (6)	0.064 (2)	0.31 (8)
C3	0.2250 (6)	0.6736 (4)	0.2436 (5)	0.065 (2)	0.70 (8)
N3	0.2810 (7)	0.6341 (4)	0.1838 (5)	0.072 (2)	0.70 (8)
C3N	0.2250 (6)	0.6736 (4)	0.2436 (5)	0.065 (2)	0.30 (8)
N3C	0.2810 (7)	0.6341 (4)	0.1838 (5)	0.072 (2)	0.30 (8)
C4	0.2166 (6)	0.1360 (4)	0.3143 (5)	0.070 (2)	0.65 (8)
N4	0.2754 (7)	0.1768 (4)	0.2599 (5)	0.067 (2)	0.65 (8)
C4N	0.2166 (6)	0.1360 (4)	0.3143 (5)	0.070 (2)	0.35 (8)
N4C	0.2754 (7)	0.1768 (4)	0.2599 (5)	0.067 (2)	0.35 (8)
C5	0.3725 (6)	0.4402 (5)	0.1119 (6)	0.0741 (19)	0.5
N5	0.3719 (6)	0.3657 (5)	0.1318 (5)	0.0722 (18)	0.5
C5N	0.3725 (6)	0.4402 (5)	0.1119 (6)	0.0741 (19)	0.5
N5C	0.3719 (6)	0.3657 (5)	0.1318 (5)	0.0722 (18)	0.5
C6	0.4684 (7)	0.6317 (5)	−0.0145 (6)	0.068 (3)	0.79 (8)
N6	0.5232 (6)	0.6729 (4)	−0.0720 (5)	0.073 (2)	0.79 (8)
C6N	0.4684 (7)	0.6317 (5)	−0.0145 (6)	0.068 (3)	0.21 (8)
N6C	0.5232 (6)	0.6729 (4)	−0.0720 (5)	0.073 (2)	0.21 (8)
O11	0.2742 (4)	0.3548 (3)	0.7144 (3)	0.0645 (11)	
C12	0.1803 (7)	0.2945 (6)	0.7398 (6)	0.087 (2)	
H12A	0.193103	0.237710	0.705079	0.130*	
H12B	0.103570	0.318424	0.714813	0.130*	
C13	0.1774 (6)	0.2804 (6)	0.8570 (6)	0.0756 (19)	
H13	0.151893	0.336224	0.889999	0.091*	
N14	0.3041 (5)	0.2619 (5)	0.8902 (5)	0.086 (2)	
H14A	0.323150	0.205475	0.875983	0.103*	
H14B	0.314170	0.270814	0.959086	0.103*	
C15	0.0893 (8)	0.2057 (8)	0.8872 (8)	0.122 (4)	
H15A	0.010376	0.218608	0.856436	0.183*	
H15B	0.116589	0.149459	0.858193	0.183*	
C16	0.0792 (12)	0.1963 (12)	1.0034 (10)	0.186 (7)	
H16A	0.023536	0.149157	1.018496	0.279*	
H16B	0.156787	0.182288	1.034068	0.279*	
H16C	0.050601	0.251419	1.032311	0.279*	
O21	0.4974 (4)	0.4360 (3)	0.7441 (4)	0.0777 (14)	
C22	0.6035 (6)	0.4630 (5)	0.8017 (6)	0.0705 (18)	
H22A	0.584489	0.511926	0.848575	0.106*	
H22B	0.663782	0.483664	0.753506	0.106*	
C23	0.6513 (5)	0.3858 (4)	0.8637 (5)	0.0548 (15)	
H23	0.685262	0.343004	0.814279	0.066*	
N24	0.5486 (5)	0.3439 (5)	0.9111 (5)	0.0836 (18)	
H24A	0.530199	0.373036	0.969548	0.100*	
H24B	0.566803	0.287729	0.928435	0.100*	
C25A	0.7491 (7)	0.4097 (6)	0.9434 (6)	0.087 (2)	0.615 (19)
H25A	0.715832	0.452718	0.991801	0.131*	0.615 (19)
H25B	0.768278	0.356241	0.983695	0.131*	0.615 (19)
C26A	0.8629 (12)	0.4466 (11)	0.9055 (12)	0.105 (6)	0.615 (19)
H26A	0.916415	0.458570	0.964309	0.158*	0.615 (19)
H26B	0.899646	0.404146	0.859638	0.158*	0.615 (19)
H26C	0.846953	0.501075	0.867783	0.158*	0.615 (19)
C25B	0.7491 (7)	0.4097 (6)	0.9434 (6)	0.087 (2)	0.385 (19)
H25C	0.812559	0.441743	0.908386	0.131*	0.385 (19)
H25D	0.716001	0.449316	0.995693	0.131*	0.385 (19)
C26B	0.800 (3)	0.333 (3)	0.995 (3)	0.183 (19)	0.385 (19)
H26D	0.861208	0.351890	1.044754	0.275*	0.385 (19)
H26E	0.737698	0.301833	1.031250	0.275*	0.385 (19)
H26F	0.834263	0.294260	0.943935	0.275*	0.385 (19)
O31	0.4789 (3)	0.5107 (4)	0.5782 (3)	0.0656 (11)	
H31	0.463 (3)	0.482 (4)	0.629 (2)	0.098*	
C32	0.5749 (7)	0.4743 (6)	0.5192 (6)	0.088 (3)	
H32A	0.567285	0.409806	0.515361	0.132*	
H32B	0.651678	0.488503	0.553397	0.132*	
C33	0.5695 (7)	0.5132 (7)	0.4117 (6)	0.090 (2)	
H33	0.588182	0.577133	0.416693	0.108*	
N34	0.4426 (5)	0.5031 (5)	0.3742 (4)	0.0810 (16)	
H34A	0.433250	0.450112	0.343149	0.097*	
H34B	0.425159	0.545258	0.326937	0.097*	
C35	0.6643 (9)	0.4674 (11)	0.3387 (8)	0.143 (5)	
H35A	0.741267	0.463956	0.376384	0.214*	
H35B	0.638242	0.406805	0.323281	0.214*	
C36	0.6806 (13)	0.5143 (16)	0.2409 (11)	0.211 (8)	
H36A	0.739051	0.483414	0.200114	0.316*	
H36B	0.605266	0.516790	0.202262	0.316*	
H36C	0.708348	0.573973	0.255395	0.316*	
O41	0.3128 (6)	0.6792 (4)	0.5131 (5)	0.0905 (16)	
H41	0.389 (2)	0.678 (6)	0.522 (4)	0.08 (3)*	
C42	0.2553 (9)	0.6937 (6)	0.6109 (7)	0.092 (2)	
H42A	0.170783	0.706689	0.597084	0.138*	
H42B	0.291188	0.745625	0.645029	0.138*	
C43	0.2652 (7)	0.6162 (5)	0.6833 (6)	0.0704 (18)	
H43	0.349305	0.610567	0.707766	0.084*	
N44	0.2308 (5)	0.5340 (3)	0.6244 (4)	0.0595 (13)	
H44A	0.153248	0.537659	0.605269	0.071*	
H44B	0.239458	0.487169	0.667042	0.071*	
C45	0.1859 (9)	0.6301 (6)	0.7794 (7)	0.095 (2)	
H45A	0.214013	0.682983	0.816758	0.142*	
H45B	0.104046	0.641692	0.754587	0.142*	
C46	0.1839 (11)	0.5535 (8)	0.8555 (8)	0.127 (4)	
H46A	0.132451	0.568038	0.912492	0.190*	
H46B	0.153872	0.500987	0.820125	0.190*	
H46C	0.264076	0.542367	0.882430	0.190*	
O51	0.2942 (4)	0.3516 (3)	0.5143 (3)	0.0560 (9)	
H51	0.291 (3)	0.356 (3)	0.5790 (19)	0.027 (12)*	
C52	0.1759 (5)	0.3430 (4)	0.4693 (5)	0.0578 (14)	
H52A	0.117561	0.363766	0.519200	0.087*	
H52B	0.159421	0.280502	0.454484	0.087*	
C53	0.1630 (5)	0.3962 (4)	0.3695 (5)	0.0533 (14)	
H53	0.226567	0.377331	0.322218	0.064*	
N54	0.1840 (5)	0.4926 (3)	0.3971 (4)	0.0601 (14)	
H54A	0.118635	0.513874	0.427577	0.072*	
H54B	0.194089	0.523440	0.338158	0.072*	
C55	0.0431 (7)	0.3810 (6)	0.3142 (6)	0.082 (2)	
H55A	0.034408	0.317527	0.300018	0.123*	
H55B	−0.020229	0.397982	0.361328	0.123*	
C56	0.0252 (9)	0.4310 (8)	0.2130 (7)	0.112 (3)	
H56A	−0.052989	0.417512	0.183150	0.169*	
H56B	0.031257	0.494045	0.226158	0.169*	
H56C	0.085993	0.413444	0.164737	0.169*	
O1	0.4541 (8)	0.2308 (8)	0.4517 (12)	0.197 (5)	
H1A	0.423 (13)	0.282 (4)	0.457 (14)	0.237*	
H1B	0.396 (9)	0.195 (8)	0.446 (15)	0.237*	
O2	0.4949 (10)	0.2175 (9)	0.6993 (9)	0.186 (4)	
H2A	0.493 (13)	0.235 (13)	0.632 (5)	0.224*	
H2B	0.574 (4)	0.224 (12)	0.710 (11)	0.224*	

### Poly[tris(2-aminobutan-1-ol)copper(II) [hexakis-µ2-cyanido-κ12C:N-tetracopper(I)] bis(2-aminobutan-1-olato)aquacopper(II) monohydrate] Atomic displacement parameters (Å^2^)

**Table:**

	*U*^11^	*U*^22^	*U*^33^	*U*^12^	*U*^13^	*U*^23^
Cu1	0.0717 (5)	0.0490 (4)	0.0752 (5)	−0.0008 (4)	0.0231 (4)	0.0030 (4)
Cu2	0.0585 (5)	0.0502 (4)	0.0764 (5)	0.0024 (4)	0.0170 (4)	−0.0054 (4)
Cu3	0.0797 (6)	0.0571 (5)	0.0638 (5)	−0.0017 (4)	0.0217 (4)	0.0020 (4)
Cu4	0.0902 (6)	0.0590 (5)	0.0625 (5)	0.0025 (4)	0.0238 (4)	0.0030 (4)
Cu5	0.0527 (4)	0.0638 (5)	0.0564 (4)	−0.0085 (4)	0.0025 (3)	0.0137 (4)
Cu6	0.0534 (4)	0.0505 (4)	0.0588 (4)	−0.0034 (3)	−0.0018 (3)	0.0070 (3)
C1	0.063 (4)	0.059 (5)	0.079 (4)	−0.003 (3)	0.022 (3)	0.003 (3)
N1	0.064 (4)	0.052 (5)	0.078 (4)	−0.004 (3)	0.022 (3)	−0.004 (3)
C1N	0.063 (4)	0.059 (5)	0.079 (4)	−0.003 (3)	0.022 (3)	0.003 (3)
N1C	0.064 (4)	0.052 (5)	0.078 (4)	−0.004 (3)	0.022 (3)	−0.004 (3)
C2	0.059 (4)	0.045 (3)	0.082 (4)	−0.008 (3)	0.028 (3)	−0.007 (3)
N2	0.062 (4)	0.045 (4)	0.087 (5)	−0.011 (3)	0.024 (4)	0.002 (3)
C2N	0.059 (4)	0.045 (3)	0.082 (4)	−0.008 (3)	0.028 (3)	−0.007 (3)
N2C	0.062 (4)	0.045 (4)	0.087 (5)	−0.011 (3)	0.024 (4)	0.002 (3)
C3	0.080 (5)	0.042 (4)	0.074 (5)	−0.002 (3)	0.024 (4)	0.008 (3)
N3	0.099 (5)	0.047 (3)	0.073 (4)	0.003 (3)	0.037 (4)	0.004 (3)
C3N	0.080 (5)	0.042 (4)	0.074 (5)	−0.002 (3)	0.024 (4)	0.008 (3)
N3C	0.099 (5)	0.047 (3)	0.073 (4)	0.003 (3)	0.037 (4)	0.004 (3)
C4	0.094 (5)	0.039 (3)	0.079 (4)	−0.001 (3)	0.038 (4)	−0.010 (3)
N4	0.091 (5)	0.046 (4)	0.066 (4)	0.007 (3)	0.032 (4)	−0.004 (3)
C4N	0.094 (5)	0.039 (3)	0.079 (4)	−0.001 (3)	0.038 (4)	−0.010 (3)
N4C	0.091 (5)	0.046 (4)	0.066 (4)	0.007 (3)	0.032 (4)	−0.004 (3)
C5	0.087 (5)	0.061 (5)	0.075 (4)	−0.001 (3)	0.032 (4)	0.001 (4)
N5	0.094 (5)	0.055 (5)	0.069 (4)	0.004 (3)	0.031 (3)	−0.003 (3)
C5N	0.087 (5)	0.061 (5)	0.075 (4)	−0.001 (3)	0.032 (4)	0.001 (4)
N5C	0.094 (5)	0.055 (5)	0.069 (4)	0.004 (3)	0.031 (3)	−0.003 (3)
C6	0.085 (5)	0.060 (4)	0.062 (4)	−0.002 (4)	0.025 (4)	−0.012 (3)
N6	0.090 (5)	0.059 (4)	0.072 (4)	−0.006 (3)	0.037 (4)	−0.005 (3)
C6N	0.085 (5)	0.060 (4)	0.062 (4)	−0.002 (4)	0.025 (4)	−0.012 (3)
N6C	0.090 (5)	0.059 (4)	0.072 (4)	−0.006 (3)	0.037 (4)	−0.005 (3)
O11	0.056 (2)	0.075 (3)	0.063 (2)	−0.013 (2)	0.0006 (18)	0.017 (2)
C12	0.063 (4)	0.113 (6)	0.083 (5)	−0.030 (4)	−0.010 (4)	0.035 (5)
C13	0.057 (4)	0.090 (5)	0.080 (5)	−0.015 (4)	0.005 (3)	0.021 (4)
N14	0.073 (4)	0.108 (5)	0.075 (4)	−0.026 (3)	−0.009 (3)	0.034 (4)
C15	0.078 (6)	0.163 (10)	0.125 (7)	−0.050 (6)	0.005 (5)	0.050 (7)
C16	0.152 (11)	0.261 (19)	0.145 (9)	−0.095 (12)	0.027 (9)	0.079 (11)
O21	0.070 (3)	0.083 (3)	0.080 (3)	−0.026 (2)	−0.013 (2)	0.034 (3)
C22	0.069 (4)	0.059 (4)	0.083 (5)	−0.015 (3)	−0.008 (4)	0.007 (4)
C23	0.056 (4)	0.052 (4)	0.057 (3)	−0.005 (3)	0.007 (3)	−0.003 (3)
N24	0.071 (4)	0.106 (5)	0.073 (4)	−0.029 (4)	−0.009 (3)	0.037 (4)
C25A	0.073 (5)	0.103 (6)	0.085 (5)	−0.020 (4)	−0.009 (4)	0.006 (4)
C26A	0.066 (8)	0.143 (13)	0.106 (11)	−0.030 (8)	−0.031 (7)	0.024 (10)
C25B	0.073 (5)	0.103 (6)	0.085 (5)	−0.020 (4)	−0.009 (4)	0.006 (4)
C26B	0.20 (3)	0.15 (2)	0.19 (3)	−0.01 (2)	−0.14 (3)	0.01 (2)
O31	0.049 (2)	0.084 (3)	0.063 (2)	−0.008 (2)	0.0015 (19)	0.021 (3)
C32	0.053 (4)	0.133 (8)	0.079 (5)	0.003 (4)	0.006 (4)	0.022 (5)
C33	0.069 (4)	0.122 (6)	0.079 (5)	−0.026 (5)	0.012 (4)	0.008 (5)
N34	0.076 (4)	0.102 (5)	0.066 (3)	−0.001 (4)	0.004 (3)	0.015 (3)
C35	0.079 (6)	0.240 (16)	0.111 (8)	0.006 (8)	0.029 (6)	0.028 (8)
C36	0.159 (12)	0.33 (2)	0.145 (11)	−0.043 (15)	0.058 (10)	0.041 (13)
O41	0.105 (5)	0.073 (3)	0.093 (4)	−0.018 (3)	−0.009 (3)	0.007 (3)
C42	0.116 (7)	0.062 (5)	0.099 (6)	−0.001 (4)	−0.007 (5)	−0.015 (4)
C43	0.073 (4)	0.062 (4)	0.076 (4)	−0.002 (3)	−0.006 (3)	−0.017 (3)
N44	0.059 (3)	0.056 (3)	0.063 (3)	0.001 (2)	−0.001 (2)	−0.004 (2)
C45	0.108 (6)	0.089 (6)	0.087 (5)	0.012 (5)	0.006 (5)	−0.022 (4)
C46	0.147 (9)	0.134 (9)	0.100 (7)	0.007 (7)	0.033 (7)	−0.009 (6)
O51	0.060 (2)	0.052 (2)	0.056 (2)	0.005 (2)	0.0057 (18)	0.003 (2)
C52	0.058 (3)	0.048 (3)	0.068 (3)	−0.005 (3)	0.011 (3)	−0.008 (3)
C53	0.054 (3)	0.047 (3)	0.059 (3)	0.005 (3)	0.009 (3)	−0.004 (3)
N54	0.064 (3)	0.046 (3)	0.070 (3)	0.009 (2)	−0.009 (3)	0.000 (2)
C55	0.071 (4)	0.088 (5)	0.086 (5)	−0.005 (4)	−0.010 (4)	−0.017 (4)
C56	0.100 (7)	0.150 (10)	0.086 (6)	0.009 (6)	−0.030 (5)	−0.008 (5)
O1	0.133 (7)	0.163 (8)	0.291 (12)	0.067 (6)	−0.082 (8)	−0.123 (9)
O2	0.173 (9)	0.171 (10)	0.213 (10)	0.032 (7)	−0.049 (8)	−0.058 (9)

### Poly[tris(2-aminobutan-1-ol)copper(II) [hexakis-µ2-cyanido-κ12C:N-tetracopper(I)] bis(2-aminobutan-1-olato)aquacopper(II) monohydrate] Geometric parameters (Å, º)

**Table:**

Cu1—C3	1.904 (7)	C26A—H26C	0.9600
Cu1—C1	1.930 (7)	C25B—C26B	1.43 (3)
Cu1—C2^i^	1.949 (6)	C25B—H25C	0.9700
Cu2—N2	1.912 (7)	C25B—H25D	0.9700
Cu2—N1^ii^	1.915 (7)	C26B—H26D	0.9600
Cu2—C4	1.954 (7)	C26B—H26E	0.9600
Cu3—C6	1.912 (8)	C26B—H26F	0.9600
Cu3—C5	1.922 (8)	O31—C32	1.427 (9)
Cu3—N3	1.947 (7)	O31—H31	0.80 (3)
Cu4—N4	1.897 (7)	C32—C33	1.486 (11)
Cu4—N5	1.929 (8)	C32—H32A	0.9700
Cu4—N6^iii^	1.945 (7)	C32—H32B	0.9700
Cu5—O21	1.901 (4)	C33—N34	1.483 (9)
Cu5—O11	1.904 (4)	C33—C35	1.578 (14)
Cu5—N14	1.984 (6)	C33—H33	0.9800
Cu5—N24	1.994 (5)	N34—H34A	0.8900
Cu5—O2	2.582 (12)	N34—H34B	0.8900
Cu6—O31	1.956 (4)	C35—C36	1.443 (16)
Cu6—N34	2.015 (6)	C35—H35A	0.9700
Cu6—N54	2.025 (5)	C35—H35B	0.9700
Cu6—N44	2.027 (5)	C36—H36A	0.9600
Cu6—O51	2.453 (5)	C36—H36B	0.9600
Cu6—O41	2.508 (6)	C36—H36C	0.9600
C1—N1	1.135 (8)	O41—C42	1.429 (10)
C2—N2	1.150 (8)	O41—H41	0.86 (3)
C3—N3	1.158 (8)	C42—C43	1.483 (11)
C4—N4	1.141 (8)	C42—H42A	0.9700
C5—N5	1.142 (8)	C42—H42B	0.9700
C6—N6	1.144 (8)	C43—N44	1.485 (8)
O11—C12	1.422 (8)	C43—C45	1.538 (11)
C12—C13	1.506 (10)	C43—H43	0.9800
C12—H12A	0.9700	N44—H44A	0.8900
C12—H12B	0.9700	N44—H44B	0.8900
C13—N14	1.484 (9)	C45—C46	1.501 (13)
C13—C15	1.540 (11)	C45—H45A	0.9700
C13—H13	0.9800	C45—H45B	0.9700
N14—H14A	0.8900	C46—H46A	0.9600
N14—H14B	0.8900	C46—H46B	0.9600
C15—C16	1.492 (14)	C46—H46C	0.9600
C15—H15A	0.9700	O51—C52	1.424 (7)
C15—H15B	0.9700	O51—H51	0.83 (2)
C16—H16A	0.9600	C52—C53	1.503 (9)
C16—H16B	0.9600	C52—H52A	0.9700
C16—H16C	0.9600	C52—H52B	0.9700
O21—C22	1.430 (8)	C53—N54	1.500 (8)
C22—C23	1.489 (9)	C53—C55	1.506 (9)
C22—H22A	0.9700	C53—H53	0.9800
C22—H22B	0.9700	N54—H54A	0.8900
C23—N24	1.447 (8)	N54—H54B	0.8900
C23—C25B	1.509 (10)	C55—C56	1.498 (12)
C23—C25A	1.509 (10)	C55—H55A	0.9700
C23—H23	0.9800	C55—H55B	0.9700
N24—H24A	0.8900	C56—H56A	0.9600
N24—H24B	0.8900	C56—H56B	0.9600
C25A—C26A	1.471 (15)	C56—H56C	0.9600
C25A—H25A	0.9700	O1—H1A	0.84 (3)
C25A—H25B	0.9700	O1—H1B	0.84 (3)
C26A—H26A	0.9600	O2—H2A	0.90 (3)
C26A—H26B	0.9600	O2—H2B	0.89 (3)
			
C3—Cu1—C1	128.4 (2)	C26B—C25B—H25C	109.0
C3—Cu1—C2^i^	117.1 (3)	C23—C25B—H25C	109.0
C1—Cu1—C2^i^	114.3 (2)	C26B—C25B—H25D	109.0
N2—Cu2—N1^ii^	126.2 (2)	C23—C25B—H25D	109.0
N2—Cu2—C4	117.2 (2)	H25C—C25B—H25D	107.8
N1^ii^—Cu2—C4	116.6 (2)	C25B—C26B—H26D	109.5
C6—Cu3—C5	129.4 (3)	C25B—C26B—H26E	109.5
C6—Cu3—N3	118.2 (3)	H26D—C26B—H26E	109.5
C5—Cu3—N3	112.3 (3)	C25B—C26B—H26F	109.5
N4—Cu4—N5	128.5 (3)	H26D—C26B—H26F	109.5
N4—Cu4—N6^iii^	119.3 (3)	H26E—C26B—H26F	109.5
N5—Cu4—N6^iii^	111.8 (3)	C32—O31—Cu6	110.9 (4)
O21—Cu5—O11	93.70 (19)	C32—O31—H31	114 (3)
O21—Cu5—N14	173.1 (3)	Cu6—O31—H31	104 (3)
O11—Cu5—N14	85.8 (2)	O31—C32—C33	108.8 (7)
O21—Cu5—N24	83.0 (2)	O31—C32—H32A	109.9
O11—Cu5—N24	176.0 (3)	C33—C32—H32A	109.9
N14—Cu5—N24	97.2 (2)	O31—C32—H32B	109.9
O21—Cu5—O2	94.2 (3)	C33—C32—H32B	109.9
O11—Cu5—O2	89.9 (3)	H32A—C32—H32B	108.3
N14—Cu5—O2	92.7 (4)	N34—C33—C32	105.7 (6)
N24—Cu5—O2	92.6 (3)	N34—C33—C35	114.1 (7)
O31—Cu6—N34	82.5 (2)	C32—C33—C35	111.1 (8)
O31—Cu6—N54	169.3 (2)	N34—C33—H33	108.6
N34—Cu6—N54	91.7 (2)	C32—C33—H33	108.6
O31—Cu6—N44	91.45 (19)	C35—C33—H33	108.6
N34—Cu6—N44	172.1 (3)	C33—N34—Cu6	111.0 (4)
N54—Cu6—N44	95.1 (2)	C33—N34—H34A	109.4
O31—Cu6—O51	93.57 (19)	Cu6—N34—H34A	109.4
N34—Cu6—O51	96.3 (2)	C33—N34—H34B	109.4
N54—Cu6—O51	78.11 (17)	Cu6—N34—H34B	109.4
N44—Cu6—O51	89.00 (17)	H34A—N34—H34B	108.0
O31—Cu6—O41	91.4 (2)	C36—C35—C33	113.4 (13)
N34—Cu6—O41	100.9 (3)	C36—C35—H35A	108.9
N54—Cu6—O41	98.56 (19)	C33—C35—H35A	108.9
N44—Cu6—O41	74.1 (2)	C36—C35—H35B	108.9
O51—Cu6—O41	162.51 (17)	C33—C35—H35B	108.9
N1—C1—Cu1	178.6 (6)	H35A—C35—H35B	107.7
C1—N1—Cu2^iv^	175.5 (6)	C35—C36—H36A	109.5
N2—C2—Cu1^v^	170.1 (6)	C35—C36—H36B	109.5
C2—N2—Cu2	175.7 (7)	H36A—C36—H36B	109.5
N3—C3—Cu1	178.4 (5)	C35—C36—H36C	109.5
C3—N3—Cu3	178.8 (6)	H36A—C36—H36C	109.5
N4—C4—Cu2	178.5 (6)	H36B—C36—H36C	109.5
C4—N4—Cu4	177.0 (6)	C42—O41—Cu6	105.5 (5)
N5—C5—Cu3	177.0 (7)	C42—O41—H41	111 (3)
C5—N5—Cu4	179.3 (6)	Cu6—O41—H41	85 (6)
N6—C6—Cu3	177.6 (6)	O41—C42—C43	113.2 (7)
C6—N6—Cu4^vi^	175.7 (7)	O41—C42—H42A	108.9
C12—O11—Cu5	111.5 (4)	C43—C42—H42A	108.9
O11—C12—C13	110.4 (6)	O41—C42—H42B	108.9
O11—C12—H12A	109.6	C43—C42—H42B	108.9
C13—C12—H12A	109.6	H42A—C42—H42B	107.7
O11—C12—H12B	109.6	C42—C43—N44	108.6 (6)
C13—C12—H12B	109.6	C42—C43—C45	110.7 (7)
H12A—C12—H12B	108.1	N44—C43—C45	111.6 (6)
N14—C13—C12	105.3 (6)	C42—C43—H43	108.6
N14—C13—C15	113.4 (7)	N44—C43—H43	108.6
C12—C13—C15	112.3 (7)	C45—C43—H43	108.6
N14—C13—H13	108.6	C43—N44—Cu6	113.9 (4)
C12—C13—H13	108.6	C43—N44—H44A	108.8
C15—C13—H13	108.6	Cu6—N44—H44A	108.8
C13—N14—Cu5	107.1 (4)	C43—N44—H44B	108.8
C13—N14—H14A	110.3	Cu6—N44—H44B	108.8
Cu5—N14—H14A	110.3	H44A—N44—H44B	107.7
C13—N14—H14B	110.3	C46—C45—C43	115.3 (7)
Cu5—N14—H14B	110.3	C46—C45—H45A	108.4
H14A—N14—H14B	108.5	C43—C45—H45A	108.4
C16—C15—C13	112.4 (9)	C46—C45—H45B	108.4
C16—C15—H15A	109.1	C43—C45—H45B	108.4
C13—C15—H15A	109.1	H45A—C45—H45B	107.5
C16—C15—H15B	109.1	C45—C46—H46A	109.5
C13—C15—H15B	109.1	C45—C46—H46B	109.5
H15A—C15—H15B	107.9	H46A—C46—H46B	109.5
C15—C16—H16A	109.5	C45—C46—H46C	109.5
C15—C16—H16B	109.5	H46A—C46—H46C	109.5
H16A—C16—H16B	109.5	H46B—C46—H46C	109.5
C15—C16—H16C	109.5	C52—O51—Cu6	101.1 (3)
H16A—C16—H16C	109.5	C52—O51—H51	110 (2)
H16B—C16—H16C	109.5	Cu6—O51—H51	92 (4)
C22—O21—Cu5	114.1 (4)	O51—C52—C53	111.0 (5)
O21—C22—C23	109.3 (5)	O51—C52—H52A	109.4
O21—C22—H22A	109.8	C53—C52—H52A	109.4
C23—C22—H22A	109.8	O51—C52—H52B	109.4
O21—C22—H22B	109.8	C53—C52—H52B	109.4
C23—C22—H22B	109.8	H52A—C52—H52B	108.0
H22A—C22—H22B	108.3	N54—C53—C52	107.5 (5)
N24—C23—C22	106.4 (5)	N54—C53—C55	112.6 (5)
N24—C23—C25B	112.6 (6)	C52—C53—C55	112.2 (5)
C22—C23—C25B	114.4 (6)	N54—C53—H53	108.1
N24—C23—C25A	112.6 (6)	C52—C53—H53	108.1
C22—C23—C25A	114.4 (6)	C55—C53—H53	108.1
N24—C23—H23	107.7	C53—N54—Cu6	113.7 (4)
C22—C23—H23	107.7	C53—N54—H54A	108.8
C25A—C23—H23	107.7	Cu6—N54—H54A	108.8
C23—N24—Cu5	110.2 (4)	C53—N54—H54B	108.8
C23—N24—H24A	109.6	Cu6—N54—H54B	108.8
Cu5—N24—H24A	109.6	H54A—N54—H54B	107.7
C23—N24—H24B	109.6	C56—C55—C53	114.9 (7)
Cu5—N24—H24B	109.6	C56—C55—H55A	108.5
H24A—N24—H24B	108.1	C53—C55—H55A	108.5
C26A—C25A—C23	118.5 (8)	C56—C55—H55B	108.5
C26A—C25A—H25A	107.7	C53—C55—H55B	108.5
C23—C25A—H25A	107.7	H55A—C55—H55B	107.5
C26A—C25A—H25B	107.7	C55—C56—H56A	109.5
C23—C25A—H25B	107.7	C55—C56—H56B	109.5
H25A—C25A—H25B	107.1	H56A—C56—H56B	109.5
C25A—C26A—H26A	109.5	C55—C56—H56C	109.5
C25A—C26A—H26B	109.5	H56A—C56—H56C	109.5
H26A—C26A—H26B	109.5	H56B—C56—H56C	109.5
C25A—C26A—H26C	109.5	H1A—O1—H1B	106 (5)
H26A—C26A—H26C	109.5	Cu5—O2—H2A	108 (10)
H26B—C26A—H26C	109.5	Cu5—O2—H2B	103 (10)
C26B—C25B—C23	112.8 (14)	H2A—O2—H2B	96 (4)
			
Cu5—O11—C12—C13	31.8 (8)	O31—C32—C33—C35	−173.5 (8)
O11—C12—C13—N14	−48.1 (9)	C32—C33—N34—Cu6	32.6 (9)
O11—C12—C13—C15	−171.9 (8)	C35—C33—N34—Cu6	155.1 (7)
C12—C13—N14—Cu5	41.0 (7)	N34—C33—C35—C36	72.6 (14)
C15—C13—N14—Cu5	164.1 (7)	C32—C33—C35—C36	−167.9 (11)
N14—C13—C15—C16	65.2 (13)	Cu6—O41—C42—C43	21.0 (8)
C12—C13—C15—C16	−175.6 (10)	O41—C42—C43—N44	−48.5 (9)
Cu5—O21—C22—C23	30.5 (7)	O41—C42—C43—C45	−171.4 (7)
O21—C22—C23—N24	−44.1 (8)	C42—C43—N44—Cu6	56.3 (7)
O21—C22—C23—C25B	−169.0 (6)	C45—C43—N44—Cu6	178.6 (5)
O21—C22—C23—C25A	−169.0 (6)	C42—C43—C45—C46	176.9 (8)
C22—C23—N24—Cu5	38.3 (7)	N44—C43—C45—C46	55.8 (10)
C25B—C23—N24—Cu5	164.4 (6)	Cu6—O51—C52—C53	43.5 (5)
C25A—C23—N24—Cu5	164.4 (6)	O51—C52—C53—N54	−61.6 (6)
N24—C23—C25A—C26A	175.4 (10)	O51—C52—C53—C55	174.1 (5)
C22—C23—C25A—C26A	−62.9 (12)	C52—C53—N54—Cu6	45.2 (5)
N24—C23—C25B—C26B	63 (2)	C55—C53—N54—Cu6	169.3 (5)
C22—C23—C25B—C26B	−175 (2)	N54—C53—C55—C56	59.6 (9)
Cu6—O31—C32—C33	44.0 (8)	C52—C53—C55—C56	−179.0 (7)
O31—C32—C33—N34	−49.2 (10)		

Symmetry codes: (i) −*x*, *y*+1/2, −*z*+1; (ii) *x*, *y*−1, *z*; (iii) −*x*+1, *y*−1/2, −*z*; (iv) *x*, *y*+1, *z*; (v) −*x*, *y*−1/2, −*z*+1; (vi) −*x*+1, *y*+1/2, −*z*.

### Poly[tris(2-aminobutan-1-ol)copper(II) [hexakis-µ2-cyanido-κ12C:N-tetracopper(I)] bis(2-aminobutan-1-olato)aquacopper(II) monohydrate] Hydrogen-bond geometry (Å, º)

**Table:**

*D*—H···*A*	*D*—H	H···*A*	*D*···*A*	*D*—H···*A*
O31—H31···O21	0.80 (3)	1.66 (2)	2.393 (6)	152 (4)
O51—H51···O11	0.83 (2)	1.74 (3)	2.562 (6)	174 (5)
N44—H44*B*···O11	0.89	2.10	2.950 (7)	159
O41—H41···O1^vii^	0.86 (3)	1.93 (5)	2.726 (10)	154 (9)
N54—H54*A*···C2^i^	0.89	2.51	3.180 (7)	133
O1—H1*A*···O51	0.84 (3)	1.93 (10)	2.669 (9)	146 (16)
O2—H2*B*···N3^viii^	0.89 (3)	2.47 (12)	3.125 (13)	130 (13)

Symmetry codes: (i) −*x*, *y*+1/2, −*z*+1; (vii) −*x*+1, *y*+1/2, −*z*+1; (viii) −*x*+1, *y*−1/2, −*z*+1.
